# Regression of cardiac growth in kidney transplant recipients using anti-m-TOR drugs plus RAS blockers: a controlled longitudinal study

**DOI:** 10.1186/1471-2369-15-65

**Published:** 2014-04-23

**Authors:** Domingo Hernández, Pedro Ruiz-Esteban, Daniel Gaitán, Dolores Burgos, Auxiliadora Mazuecos, Rocío Collantes, Eva Briceño, Eulalia Palma, Mercedes Cabello, Miguel González-Molina, Manuel De Mora

**Affiliations:** 1 Department of Nephrology, Carlos Haya University Hospital and IBIMA, Malaga E-29010, Spain; 2 Department of Cardiology and Radiology, Carlos Haya University Hospital and IBIMA, Malaga E-29010, Spain; 3 Department of Radiology, Carlos Haya University Hospital and IBIMA, Malaga E-29010, Spain; 4 Department of Nephrology, Hospital Puerta del Mar, Cadiz E-11009, Spain

**Keywords:** Everolimus, Kidney transplantation, Left ventricular hypertrophy, Renin-angiotensin blockers, Sirolimus

## Abstract

**Background:**

Left ventricular hypertrophy (LVH) is common in kidney transplant (KT) recipients. LVH is associated with a worse outcome, though m-TOR therapy may help to revert this complication. We therefore conducted a longitudinal study to assess morphological and functional echocardiographic changes after conversion from CNI to m-TOR inhibitor drugs in nondiabetic KT patients who had previously received RAS blockers during the follow-up.

**Methods:**

We undertook a 1-year nonrandomized controlled study in 30 non-diabetic KT patients who were converted from calcineurin inhibitor (CNI) to m-TOR therapy. A control group received immunosuppressive therapy based on CNIs. Two echocardiograms were done during the follow-up.

**Results:**

Nineteen patients were switched to SRL and 11 to EVL. The m-TOR group showed a significant reduction in LVMi after 1 year (from 62 ± 22 to 55 ± 20 g/m^2.7^; *P* = 0.003, paired *t*-test). A higher proportion of patients showing LVMi reduction was observed in the m-TOR group (53.3 versus 29.3%, *P* = 0.048) at the study end. In addition, only 56% of the m-TOR patients had LVH at the study end compared to 77% of the control group (*P* = 0.047). A significant change from baseline in deceleration time in early diastole was observed in the m-TOR group compared with the control group (*P* = 0.019).

**Conclusions:**

Switching from CNI to m-TOR therapy in non-diabetic KT patients may regress LVH, independently of blood pressure changes and follow-up time. This suggests a direct non-hemodynamic effect of m-TOR drugs on cardiac mass.

## Background

Left ventricular hypertrophy (LVH) is a very prevalent clinical-pathological entity after kidney transplantation (KT), and is a significant predictor of long-term adverse KT outcome [1]. Classical and nontraditional risk factors inherent to KT may participate in its pathogenesis [2]. Thus, regression of LVH is an important therapeutic target in order to optimize survival rates in these patients.

Mammalian target of rapamycin (m-TOR) inhibitors may inhibit cardiac growth by antiproliferative effects. Indeed, inhibition of the proliferation signal with sirolimus (SRL) led to regression of pressure load-induced LVH in animal models [3-5]. In addition, nonrandomized controlled trials have shown that conversion from calcineurin inhibitors (CNI) to SRL may regress LVH in KT patients [6,7]. More recently, a randomized controlled study demonstrated that the use of everolimus (EVL) plus a reduced exposure of cyclosporine (CsA) proved effective in regressing LVH in KT recipients [8]. However, none of these studies included patients who had received renin-angiotensin system (RAS) blockers together with the m-TOR inhibitors during the first post-conversion year. RAS blockers are frequently used in KT recipients as cardio-protective and reno-protective drugs and, indeed, their use has been associated with regression of LVH after KT [9,10]. Thus, a potential additive effect on cardiac mass seems plausible when both drugs, m-TOR inhibitors and RAS blockers, are administered together in this particular population. Finally, little information has been provided about the effect of m-TOR inhibitors on Doppler-derived diastolic function [11]. Angiotensin II leads to prolonged diastolic filling [12], and changes in diastolic function might be expected when both RAS blockers and m-TOR inhibitors concur in these patients.

In consonance with these arguments, we conducted a longitudinal study to assess morphological and functional echocardiographic changes after conversion from CNI to m-TOR inhibitor drugs in nondiabetic KT patients who had previously received RAS blockers during the follow-up.

## Methods

### Design

This 1-year longitudinal nonradomized controlled study involved nondiabetic KT patients who were switched from a CNI (CsA or tacrolimus) to an immunosuppressive regimen based on SRL or EVL plus mycophenolate mofetil and steroids, in accordance with clinical practice. Thus, inclusion criteria in the cohort were: 1) clinical indication for conversion due to either biopsied chronic allograft dysfunction or non-melanoma skin cancer; 2) stable renal function (serum creatinine <2.5 mg/dL); 3) 24-hour urinary protein excretion <500 mg/day; and 4) signed informed written consent. Exclusion criteria were: 1) pre-existing lung or heart disease such as chronic respiratory disease, valvular abnormalities, ischemic heart disease and congestive heart failure; 2) impaired renal function (serum creatinine >2.6 mg/dL); or 3) proteinuria >500 mg/day.

The patients underwent two echocardiographic studies to examine the structural and functional changes in LV mass after the first post-conversion year. In accordance with our daily clinical practice in KT recipients, all the patients received RAS blockers (angiotensin-converting enzyme inhibitors or angiotensin receptor blockers) prior to inclusion in this study.

### Patients and follow-up study

A total of 46 consecutive non-diabetic KT recipients who were converted from a CNI to a m-TOR inhibitor between February 1, 2010 and January 31, 2012 were initially enrolled in this study. The main reasons for conversion were non-melanoma skin cancer (n = 30) and chronic allograft dysfunction (n = 16). Sixteen patients were excluded due to m-TOR-related side effects after conversion (n = 9) or withdrawal of consent (n = 7) during follow-up. Thus, the final cohort involved 30 patients who completed the 1-year observation period.

A control group was composed of 58 age-matched KT recipients without diabetes and with a similar time after grafting who received immunosuppressive therapy based on CNIs. All also received RAS blockers during the study period.

The goal of antihypertensive therapy was to obtain a blood pressure ≤130/80 mmHg in both groups during the study. Thus, antihypertensive agents, other than RAS blockers, were adjusted to achieve this blood pressure control during the follow-up as in standard clinical practice.

Medical record review was performed according to Spanish law with reference to clinical data confidentiality. This study was approved by the Ethics Committee of Carlos Haya University Hospital and was conducted according to the Declaration of Helsinki. Each patient gave written informed consent to participate in the study.

### Laboratory measurements

Blood sampling for the measurement of routine and other special biochemical measurements was performed before the echocardiographic studies. Titration of m-TOR drugs was tailored twice monthly in an attempt to keep EVL and SRL trough levels between 4–7 ng/mL. The daily urinary protein excretion rate was also assessed at baseline and then monthly.

### Echocardiography

Using standard methods, M-mode, two-dimensional and color flow Doppler echocardiograms were performed by a single experienced cardiologist (DG) at baseline and after 12 months in both groups, blinded to the clinical characteristics of the participants. Echocardiograms were obtained with the patient in the left decubitus position with 30° head inclination, using an ultrasonoscope system (Philips iE33) equipped with a 1- to 5- MHz versatile (X5-1) transducer. All echocardiographic measurements were undertaken following the recommendations of the American Society of Echocardiography [13]. Intraobserver variability was less than 5%.

Left ventricular end-diastolic diameter (LVEDD), posterior wall thickness (PWT), and the interventricular septum thickness (IVS) were measured at end diastole. Left ventricular mass (LVM) was defined according to the equation [14]: LVM = 0.80 × 1.04 × [(IVS + PWT + LVEDD)^3^)- LVEDD^3^] + 0.6 g. and then indexed for height^2.7^, which has been documented as the reliable indexation for patients with renal failure [15]. LVH was defined by a LVMi >49.2 g/m^2.7^ and >46.7 g/m^2.7^ in accordance with previously reported cutoff values for men and women, respectively [16]. Left ventricular relative wall thickness was calculated as (IVS + PWT)/LVEDD [17]. The percentage of fractional shortening of the left ventricle was calculated to evaluate systolic function by the formula LVEDD-LVESD/LVEDD × 100, where LVESD is left ventricular end-systolic diameter. The left ventricular ejection fraction was also assessed. The Doppler indexes measured were deceleration time of flow velocity in early diastole (DT) and left ventricular isovolumic relaxation time (LVIRT) in ms, and peak early diastolic velocity (E) and peak atrial diastolic velocity (A) in centimeters per second. In addition, the E/A ratio was also calculated.

### Outcome

The primary outcome was determined as percent change in LVMi (ΔLVMi) between the two echocardiographic studies ([baseline value – final value] × 100/baseline value).

### Statistical analysis

Data are presented as mean ± SD or median ± interquartile range. Comparisons of continuous variables between the two groups were made by means of Student’s *t* test or Mann–Whitney U test in the case of nonparametric distribution. Paired *t*-test (or Wilcoxon signed-rank test depending on distribution of data) was used for intra-group comparisons. The Chi-square test or Fisher exact test, when appropriate, were used for inter-group comparisons of categorical variables. Multiple regression analysis was performed to determine independent predictors of the final ΔLVMi from baseline. We also screened the following variables: age, gender, primary cause of kidney disease, follow-up time, use of RAS blockers, body mass index (BMI kg/m^2^), blood pressure, hemoglobin levels, renal function, baseline LVMi, and changes from baseline of blood pressure, BMI and hemoglobin levels. Co-linearity and the assumption of normality were never violated. Computations were made by SPSS 15.0 for Windows (SPSS Inc., Chicago, IL). A *P* value less than 0.05 was considered significant.

## Results

Of the 30 patients in the m-TOR group who completed the 1-year observation period, 19 were switched to SRL and 11 to EVL. No patients who ended the study period experienced acute rejection. The median time from transplantation to m-TOR therapy conversion was 64 months (interquartile range 16–105 months).

Table 1 summarizes the clinical and demographic data for the two groups. As expected, a higher baseline 24-hour urinary protein excretion was observed in the m-TOR inhibitor group because chronic allograft dysfunction was present in 16 patients prior to conversion. No significant differences were found in other clinical variables such as age, gender, cause of renal disease, blood pressure, number of antihypertensive drugs, BMI, serum creatinine, hemoglobin levels, lipid profile or time from transplantation to the end of the study. The number of baseline antihypertensive drugs was similar in the two study groups.

**Table 1:** Baseline demographic and clinical data of the two study groups

	**m-TOR group (n = 30)**	**Control group (n = 58)**	***P *** **value**
**Age (y)**	58.2 ± 13.7	54.5 ± 14	0.239
**Gender (Male/Female)**	21/9	37/21	0.560
**Cause of CKD**			
**(GN/PKD/HKD/IN/Other)**	9/4/1/3/13	23/4/6/9/16	0.370
**Time from transplant to study end (mo)**	89.7 ± 72	82 ± 30	0.574
**Systolic blood pressure (mm Hg)**	126 ± 11	130 ± 12	0.122
**Diastolic blood pressure (mm Hg)**	73 ± 9	76 ± 8	0.129
**Serum creatinine (mg/dL)**	1.56 ± 0.3	1.5 ± 0.4	0.464
**No. of antihypertensive drugs**	1.6 ± 1	1.8 ± 0.8	0.224
**Hemoglobin (g/dL)**	13 ± 1.5	13.6 ± 1.8	0.101
**Cholesterol (mg/dL)**	177 ± 31	189 ± 40	0.124
**Triglycerides (mg/dL)**	140 ± 60	165 ± 80	0.103
**Uprot (mg/24 h)**	275 ± 232	32 ± 47	0.000
**BMI (kg/m**^**2**^**)**	28 ± 4.7	28.3 ± 4.4	0.736

Values are shown as mean ± SD or absolute values. To convert creatinine in mg/dl to μmol/L, multiply by 88.4; Hemoglobin in g/dl to g/L, multiply by 10; cholesterol in mg/dL to mmol/L, multiply by 0.02586; triglycerides in mg/dL to mmol/L, multiply by 0.01129.

*Abbreviations:* IN, Interstitial nephropathy; CKD, chronic kidney disease; GN, glomerulonephritis; PKD, polycystic kidney disease; HKD, hypertensive kidney disease; IN, interstitial nephropathy; BMI, body mass index; Uprot, daily urinary protein excretion.

No significant differences were found between the two groups with regard to baseline ecochardiographic morphological data. Furthermore, the prevalence of LVH was similar among patients with and without m-TOR inhibitors (Table 2). However, the m-TOR patients showed a longer peak atrial diastolic velocity compared with the control group. As a consequence, the E/A ratio was significantly higher in the control group.

**Table 2:** Baseline morphological and functional echocardiographic data in both groups

	**m-TOR group (n = 30)**	**Control group (n = 58)**	***P *** **value**
**LAD (mm)**	38.4 ± 5.5	39.5 ± 5.5	0.379
**LVEDD (mm)**	48.2 ± 5.4	49.7 ± 6	0.259
**IVS (mm)**	12.7 ± 2.4	13 ± 2.7	0.259
**PWT (mm)**	12.3 ± 2.2	12.1 ± 2.1	0.832
**RWT**	0.52 ± 0.1	0.51 ± 0.1	0.948
**EF (%)**	69.7 ± 6	71.5 ± 8	0.272
**FS (%)**	46 ± 8.5	43 ± 7	0.102
**LVMI (g/m**^**2.7**^**)**	62 ± 22	65 ± 17	0.471
**LVH prevalence (%)**	77	86	0.259
**Peak E (cm/sec)**	0.80 ± 0.2	0.73 ± 0.2	0.099
**Peak A (cm/sec)**	0.92 ± 0.3	0.7 ± 0.2	0.000
**E/A ratio**	0.92 ± 0.3	1.1 ± 0.4	0.014
**DT (msec)**	215.5 ± 89	236 ± 64	0.673
**LVIRT (msec)**	99.3 ± 23	100.6 ± 39	0.847

Values are shown as mean ± SD or percentages.

Abbreviations: LAD, left atrial diameter; LVEDD, left ventricular end-diastolic diameter; IVS, interventricular septal thickness; PWT, posterior wall thickness; RWT, relative wall thickness; EF, left ventricular ejection fraction; FS, left ventricular fractional shortening; LVMI, left ventricular mass index; LVH, left ventricular hypertrophy; Peak E, peak early diastolic flow velocity; peak A, peak late diastolic flow velocity; E/A ratio, ratio of early to late diastolic flow; DT deceleration time of E wave; LVIRT, left ventricular isovolumetric relaxation time.

Comparisons of changes in clinical data at the end of the follow-up between the two groups are shown in Table 3. Both groups were receiving a similar proportion of other antihypertensive drugs at the study end (56 versus 65%; *P* = 0.3779), but a non-significant reduction of blood pressure was more evident in patients switched from CNI to m-TOR inhibitors. As expected, a significant increase in triglyceride levels was observed in the m-TOR group, even though 40% of the patients received lipid-lowering drugs. In addition, a significant increase in proteinuria values was also documented in patients who received m-TOR compared with the control group. Lastly, no significant differences were observed for BMI, cholesterol concentration, or hemoglobin and serum creatinine levels between the two groups.

**Table 3:** Changes in clinical parameters from baseline to 12 months in both groups

	**Δ, m-TOR group ( *P *)**	**Δ, Control group ( *P *)**	**Δ, Effect (m-TOR vs.Control), (95% CI)**	***P *** **value**
**BMI (kg/m**^**2**^**)**	1.37 ± 7	0.2 ± 5	1.1 ± 1.3	0.459
	(0.2)	(0.7)	(-1.8 to 4)	
**Systolic blood pressure (mmHg)**	-1.7 ± 12	0.98 ± 12	2.6 ± 2.7	0.324
	(0.6)	(0.4)	(-8 to 2.7)	
**Diastolic blood pressure (mmHg)**	-2.8 ± 16	0.7 ± 14.7	-3.6 ± 3.5	0.326
	(0.7)	(0.3)	(-10 to 3.6)	
**Serum creatinine (mg/dL)**	-0.44 ± 22	-0.55 ± 24	0.1 ± 5.4	0.985
	(0.7)	(0.4)	(-10.8 to 11)	
**Hemoglobin (g/dL)**	-7.6 ± 6.6	-6 ± 16	-1.7 ± 2.6	0.503
	(0.0001)	(0.002)	(-6.8 to 3.4)	
**Uprot (mg/24 h)**	-278 ± 572	-6.1 ± 98	-271.6 ± 116	0.028
	(0.003)	(0.4)	(-511 to -32)	
**Cholesterol (mg/dL)**	-12.1 ± 22	-5.9 ± 21	-6.2 ± 5.2	0.243
	(0.01)	(0.15)	(-17 to 4.3)	
**Triglycerides (mg/dL)**	-24.6 ± 58	6.3 ± 33	-31 ± 12.3	0.017
	(0.08)	(0.010)	(-56 to -5.8)	

Δ, percent increase or decrease from baseline ([baseline value – final value] x 100/baseline value). Data are expressed as mean ± SD. Values in parentheses are *P* values for the differences between final and baseline absolute values, and 95% confidence intervals for the control versus m-TOR group effect.

To convert serum creatinine in mg/dL to mol/L, multiply by 88.4; hemoglobin in g/dL to g/L, multiply by 10; cholesterol in mg/dL to mmol/L, multiply by 0.02586; triglycerides in mg/dL to mmol/L, multiply by 0.01129.

*Abbreviations:* BMI, body mass index; Uprot, daily urinary protein excretion.

**Figure 1:**
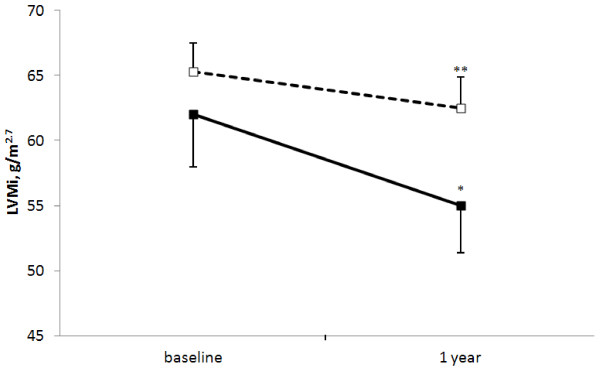
**Change in left ventricular mass index.** Change in left ventricular mass index (LVMi; mean ± SEM) in 30 renal transplant recipients on m-TOR therapy (filled squares) and 58 controls (open squares) over a 1-year observation period. *P = 0.003 and **P = 0.052 compared with baseline LVMi.

The m-TOR group showed a more significant reduction in LVMi after 1 year (from 62 ± 22 to 55 ± 20 g/m^2.7^; *P* = 0.003, paired *t*-test), resulting mainly from a significant decrease in thickness of both the IVS (12.7 ± 2.4 to 12 ± 2.2 mm; *P* = 0.002, paired *t*-test) and posterior wall (12.2 ± 2.2 to 11.3 ± 2 mm; *P* = 0.001; paired *t*-test). On the other hand, a smaller change in LVMi was documented in the control group at the study end (Figure 1 and Table 4). As a result, a higher proportion of patients showing a reduction (>10%) in LVMi was observed in the m-TOR group (53.3 versus 29.3%, *P* = 0.048) after 12 months of follow-up. In addition, at the end of the study only 56% of patients met criteria for LVH whereas 77% of the control group met these criteria (*P* = 0.047). Finally, a significant increase in the peak early diastolic velocity was only observed in the m-TOR group (0.80 ± 0.22 to 0.92 ± 0.27 cm/sec; *P* = 0.034; paired *t*-test).

**Table 4:** Changes in echocardiographic parameters from baseline to 12 months in both groups

	**Δ, m-TOR group ( *P *)**	**Δ, Control group ( *P *)**	**Δ Effect (m-TOR vs Control) (95%CI)**	***P *** **value**
**LAD (mm)**	0.90 ± 15	0.30 ± 14	0.59 ± 3.4	0.859
	(0.4)	(0.6)	(-6 to 7.3)	
**LVEDD (mm)**	45.8 ± 10	44 ± 9	1.8 ± 2.1	0.428
	(0.7)	(0.2)	(-2.5 to 5.8)	
**IVS (mm)**	5.3 ± 8.5	3.1 ± 16	2.2 ± 2.7	0.427
	(0.002)	(0.04)	(-3.1 to 7.4)	
**PWT (mm)**	7 ± 10	4.9 ± 14	2.1 ± 2.7	0.415
	(0.001)	(0.01)	(-3.1 to 7.5)	
**RWT**	4.8 ± 14	5 ± 17	0.2 ± 3.7	0.949
	(0.026)	(0.035)	(-7.1 to 7.6)	
**EF (%)**	6.6 ± 11	0.5 ± 16	6.1 ± 3.6	0.101
	(0.7)	(0.2)	(-1.2 to 13)	
**FS (%)**	-1.05 ± 21	-8 ± 25	7.1 ± 5.4	0.197
	(0.2)	(0.02)	(-3.7 to 18)	
**LVMi (g/m**^**2.7**^**)**	8.4 ± 18	3.8 ± 16	4.5 ± 3.9	0.255
	(0.003)	(0.052)	(-3.4 to 12)	
**Peak E (cm/sec)**	-19.4 ± 43	-6.4 ± 25.6	-13 ± 9	0.135
	(0.034)	(0.4)	(-30 to 4.6)	
**Peak A (cm/sec)**	-11.1 ± 33	-9.3 ± 27	-1.7 ± 7	0.808
	(0.3)	(0.1)	(-16 to 12)	
**E/A ratio**	-0.06 ± 0.34	-0.001 ± 0.34	-12.6 ± 9.8	0.429
	(0.3)	(0.9)	(-32 to 7)	
**DT (msec)**	-18.2 ± 9	-5 ± 40	-13 ± 7.8	0.019
	(0.05)	(0.8)	(-29 to 3.5)	
**LVIRT (msec)**	-10.3 ± 33	-3 ± 31	-7.1 ± 7.5	0.341
	(0.4)	(0.5)	(-22 to 8)	

Δ, percent increase or decrease from baseline ([baseline value – final value] x 100/baseline value). Values are expressed as mean ± SD; values in parentheses are *P* values for the differences between final and baseline absolute values, and 95% confidence intervals for the control versus m-TOR group effect.

Abbreviations: LAD, left atrial diameter; LVEDD, left ventricular end-diastolic diameter; IVS, interventricular septal thickness; PWT, posterior wall thickness; RWT, relative wall thickness; FS, left ventricular fractional shortening; LVMI, left ventricular mass index; Peak E, peak early diastolic flow velocity; peak A, peak late diastolic flow velocity; E/A ratio, ratio of early to late diastolic flow; DT deceleration time of E wave; LVIRT, left ventricular isovolumetric relaxation time.

Table 4 displays the changes in echocardiographic parameters from baseline to 12 months. A clinically more pronounced but non-significant change in LVMi was seen in the m-TOR group compared with the control group. Of note, a significant change in DT from baseline was observed in the m-TOR group compared with the control group. Moreover, a trend toward a greater change in peak early diastolic velocity was also documented in the m-TOR group. No other differences were observed from baseline between the two study groups.

By backward linear regression analyses, baseline LVMi (β = 0.334, *P* = 0.004) and m-TOR therapy (β = 0.236; *P* = 0.043) were significantly associated with LVMi changes, after adjusting for age, gender, blood pressure, hemoglobin level, BMI and time after grafting, all of which accounted for 40% of the total variation in ΔLVMi.

## Discussion

The most relevant finding of this prospective cohort study was that conversion from a CNI to m-TOR inhibitor is associated with marked LVH regression in non-diabetic KT recipients receiving RAS blockers, whereas only a modest LVMi change was observed in the control group. This reduction was achieved mainly by reducing the ventricular wall thickness and interventricular septum. No differences were found in terms of proteinuria, renal function, hemoglobin levels, incidence of adverse events, lipid profile or LVMi change between SRL and EVL after conversion (data not shown). As a consequence, a significantly higher proportion of patients showed a reduction in LVH in the m-TOR group compared with the control group. In addition, regression of LVH was independent of blood pressure and the post-transplant time, among other risk factors affecting LV mass. We cannot rule out, though, that substantially different hemodynamic effects between the two treatment groups (CNI versus m-TOR therapy), affecting only modestly blood pressure, could modulate LVM changes at the end of the follow-up. Indeed, non significant differences in brachial pressure between different antihypertensive regimens may lead to significant changes in LVM by increasing central aortic pressure, as previously reported [18,19]. The change in immunosuppression was based on previously reported beneficial effects of replacing CNI with m-TOR inhibitors when side effects, chronic allograft dysfunction or skin cancer occur in KT patients during follow-up, as in our study. In order to minimize coexistent abnormalities that could affect cardiac growth, we only included non-diabetic patients with an acceptable renal graft function and absence of mild proteinuria.

Our results are consistent with findings derived from animal model studies demonstrating that the use of a SRL dosage similar to that prescribed in KT was associated with regression of pressure-induced cardiac hypertrophy by means of antiproliferative mechanisms [3-5]. In addition, Paoletti et al. demonstrated in controlled studies that both SRL and EVL regress left ventricular mass in KT recipients [6-8]. However, no patient received combined treatment with m-TOR inhibitors and RAS blockers during the first 12 months post-conversion. RAS blockers are widely used in KT patients and have been associated with LVH regression, especially when the machinery of cardiac growth is activated, as reported in KT recipients [9].

Currently, CNIs are the cornerstone of immunosuppressive treatment for KT patients. These drugs may result in the development of cardiac hypertrophy and myocardial fibrosis by stimulating both circulating and local RAS. Angiotensin II activates m-TOR-p70 ribosomal S6 kinase, which regulates protein synthesis in cardiac myocytes [20,21]. In addition, m-TOR drugs may attenuate the angiotensin II-induced increase in protein synthesis by blocking phosphorylation of the p-70 ribosomal S6 protein involved in cardiac growth [20,22]. In consonance with these findings, RAS blockers are effective in reducing LVH after KT, independently of blood pressure [9,23]. In a controlled clinical trial evaluating the effects of conversion from CNIs to SRL in KT recipients, Paolleti et al. observed a significant regression of LVMi in patients treated with SRL and RAS blockers at the third year of follow-up, but RAS blockers were only prescribed after the first post-conversion year [7]. In contrast, a recent clinical trial in KT recipients comparing EVL-based versus CsA-based immunosuppression found no differences in LV mass between the two groups, but the CsA group received a significantly higher proportion of antihypertensive drugs, including RAS blockers. Furthermore, RAS blockers were not used in the EVL-group [24].

In our study, all the patients in the m-TOR group had received RAS blockers prior to conversion, according to our daily clinical practice. Thus, both types of drugs, m-TOR and RAS blockers were administered in this group throughout the whole follow-up period after conversion. Our results suggest that, in non-diabetic renal transplant recipients, a more pronounced effect of m-TOR drugs on left ventricular mass might be expected in the presence of RAS blockers. In other words, a synergistic effect on regression of LVH seems plausible when both kinds of drugs are administered in this population, especially after suppression of CNI. Whether a similar effect on cardiac mass would be observed in KT recipients on m-TOR inhibitors therapy but without additional RAS blockers cannot be determined from this study.

Myocardial interstitial fibrosis is a typical pattern associated with LVH in uremic patients [22]. m-TOR inhibitors and RAS blockers are potent antifibrotic agents [20,25]. We cannot rule out a greater response to the effect of m-TOR drugs in patients with both myocardial fibrosis and histological features of chronic allograft dysfunction. However, the fact that the control patients, who mostly received RAS blockers, showed a lesser reduction in cardiac mass with an allograft function similar to the m-TOR group after 12 months makes this unlikely.

Cardiac hypertrophy and myocardial fibrosis have been associated with CNI treatment, contributing to diastolic abnormalities and elevation of filling pressures [11]. Inhibition of m-TOR reduces cardiac growth and fibrosis. LVH reduction after conversion from CNI drugs to m-TOR therapy was accompanied by a change in passive ventricular filling pattern, as evidenced by a significant DT change from baseline. Likewise, a more pronounced change in peak early diastolic velocity was observed in the m-TOR group compared with the control group, who had a higher baseline E/A ratio. In this respect, a previous report has demonstrated that SRL improves parameters of diastolic function in heart transplant patients [11]. However, to our knowledge, no reports have previously documented diastolic function changes in KT recipients who received both m-TOR therapy and RAS blockers. Given the effects of angiotensin II on diastolic filling [12], a change in early diastolic function might occur in KT recipients with an activated RAS, as evidenced in our study. We cannot rule out that assessment of diastolic function by pulsed-wave tissue Doppler echocardiography, such as evaluation of mitral annulus velocity, could elucidate more accurately the changes in the diastolic performance than standard echocardiography. Taken together, we speculate that combined administration of m-TOR drugs and RAS blockers may lead to a reduction of both cardiac fibrosis and angiotensin-induced hypercontractility, contributing to optimizing cardiac remodeling and distensibility, as well as the diastolic filling pattern. Whether this finding is associated with a lower risk of post-transplant heart failure in the long term is uncertain.

As expected, a slight but significant increase in both the urinary protein excretion rate and lipid levels was observed in patients on m-TOR therapy, which may be clinically relevant given the association of low-grade proteinuria and hyperlipidemia with increased cardiovascular risk in KT recipients [26,27]. Nevertheless, the proportion of patients receiving statins and the number of individuals with proteinuria greater than 0.5 g/day were similar in the two groups after the one-year observation period (data not shown). In theory, this suggests that patients on m-TOR therapy should not be exposed to a higher cardiovascular risk. Future longitudinal and prospective studies will be needed to clarify the prognostic significance of these findings.

This study has some limitations, the most important of which is that it was not a blinded, randomized study. Secondly, we studied a highly selected cohort of KT recipients who presented transplant-related clinical complications such as chronic allograft dysfunction or non-melanoma skin cancer, which could limit the generalizability of our findings. In addition, our study was a single-center trial and the changes in LVM could be the effect of intrapatient variability. However, all the subjects in the final cohort completed the investigation and prospective echocardiographic examinations were performed after conversion by the same cardiologist, with an intraobserver variability lower than 5%. This, in theory, should minimize the risk of misleading measurements. Moreover, control patients, who received only CNI as the main immunosuppressive therapy, underwent two echocardiograms during the same study period, though the changes in LVH reduction were more modest as compared with the m-TOR group. Finally, regression of the mean phenomenon may have occurred in this study, but the results were adjusted for baseline LVMi as a predictor variable, which makes it unlikely.

## Conclusion

In conclusion, conversion from CNI to m-TOR therapy in non-diabetic KT recipients was associated with regression of LVH, independently of other risk factors for cardiac growth. In addition, a change in diastolic filling pattern may be achieved with this strategy, possibly linked to changes in cardiac remodeling and distensibility. Future large randomized studies are needed to determine whether m-TOR drugs should be used as first line therapy to optimize cardiac remodeling in this population.

## Abbreviations

LVH: Left ventricular hypertrophy; KT: Kidney transplant; CNI: Calcineurin inhibitors; mTOR: Mammalian target of rapamycin; SRL: Sirolimus; EVL: Everolimus; CsA: Cyclosporine; RAS: Rennin-angiotensin system; LVEDD: Left ventricular end-diastolic diameter; PWT: Posterior wall thickness; IVS: Interventricular septum thickness; LVM: Left ventricular mass; LVESD: Left ventricular end-systolic diameter; LVIRT: Left ventricular isovolumic relaxation time; E: Peak early diastolic velocity; A: Peak atrial diastolic velocity.

## Competing interests

The authors declare that they have no competing interests.

## Authors’ contributions

All the authors have contributed to the manuscript. DH wrote the manuscript, designed the study, performed statistical analyses, contributed to discussion and reviewed/edited the manuscript. PRE wrote manuscript, performed statistical analyses, contributed to discussion and reviewed/edited the manuscript. DG researched data and contributed to discussion. DB contributed to discussion and reviewed/edited the manuscript. AM wrote the manuscript, researched data and contributed to discussion. RC, EB, EP researched data and contributed to discussion. MC contributed to discussion. MGM, MM reviewed/edited the manuscript. All authors have read and approved the final version.

## Pre-publication history

The pre-publication history for this paper can be accessed here:

http://www.biomedcentral.com/1471-2369/15/65/prepub
